# Supplementary material for: Integrated analysis of the aqueous humor microbiome and lens capsule transcriptome in high myopia cataract: a pilot study
Source: Front Med (Lausanne). 2026 Jun 16;13:1845205. doi: 10.3389/fmed.2026.1845205 (PMC13314463; doi:10.3389/fmed.2026.1845205)
Supplement: Supplementary file 3 [file Data_Sheet_1.zip › 4.Meta_Statistic/C372089/Wilcoxon/Class/diff_Class_top10_boxplot.pdf]

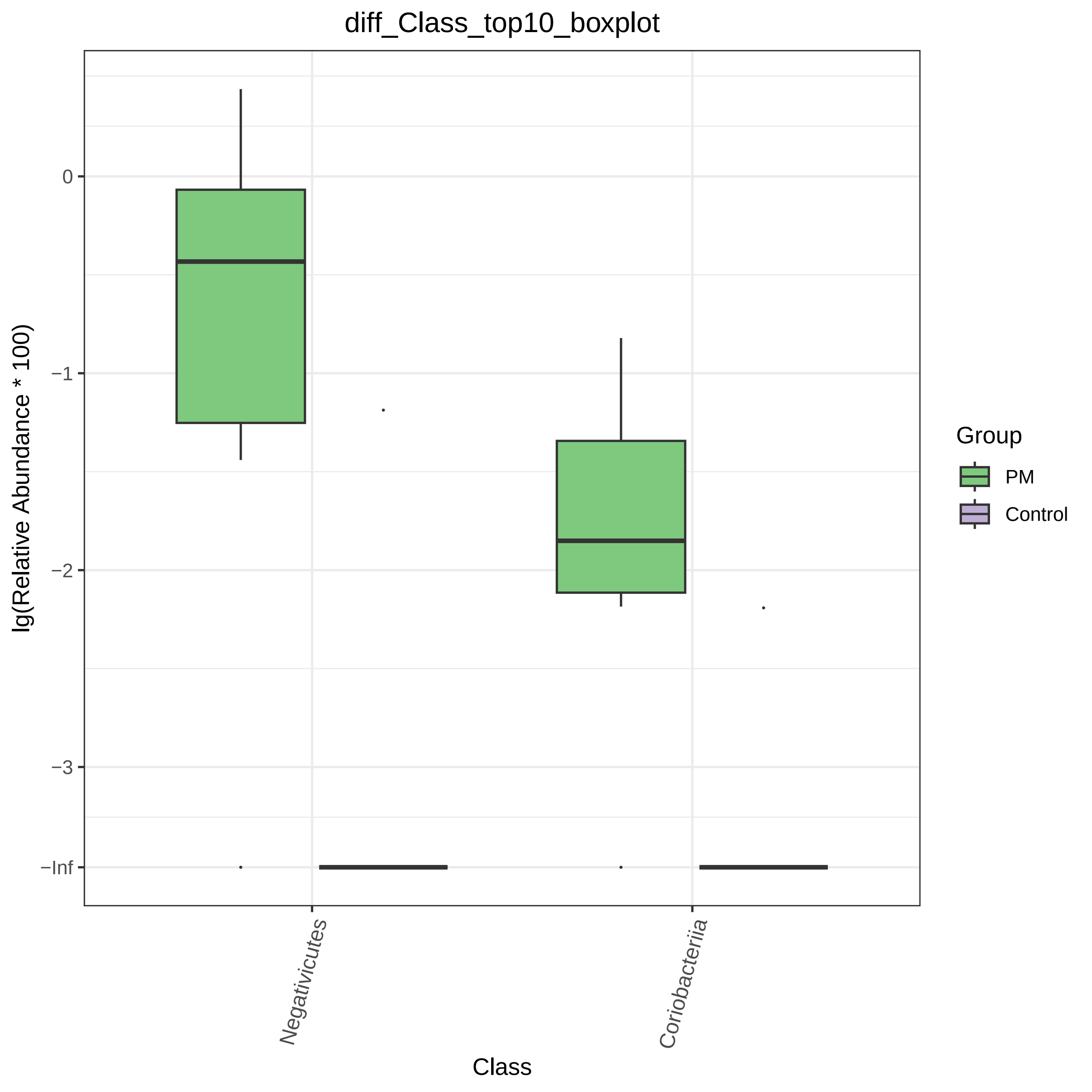

diff_Class_top10_boxplot
lg(Relative Abundance * 100)
0
−1
−2
−3
−Inf
Negativicutes
Coriobacteriia
Class
Group
PM
Control
